# Plasma cortisol activity in rats under conditions of chronic stress supplemented with resveratrol

**Published:** 2012-09-30

**Authors:** Miryam Vélez-Marín, Alejandro Hurtado Salazar, Luis F Uribe-Velásquez

**Affiliations:** a Department of Biological Sciences, School of Natural and Exact Sciences, Universidad de Caldas, Manizales, Colombia.; b Department of Production Systems, School of Agricultural Sciences, Universidad de Caldas, Manizales, Colombia.; c Department of Animal Health, School of Agricultural Sciences, Universidad de Caldas, Manizales, Colombia.

**Keywords:** Adrenocorticotropic hormone, antioxidants, phenolic compounds

## Abstract

**Objective:**

To determine the activity of cortisol in rats treated with exogenous adrenocorticotropic hormone (ACTH) and a resveratrol supplement.

**Methods:**

Forty-eight adult female rats and 16 male rats of the strain (*Rattus norvegicus*) that were three months old and with body weights ranging from 200 to 250 g for females and 300 to 350 g for males were used and kept in controlled environmental conditions: temperature of 20±2° C and light-dark cycles of 14 and 10 hours. They were fed a balanced diet and had free access to water. The rats were randomly divided into four groups: group 1 - was treated with 5 µg/kg of ACTH i.p. every twelve hours; group 2 - received the same treatment with ACTH plus a grape extract supplement (resveratrol) of 40 mg/kg; group 3 - only received grape extract (resveratrol); and group 4 - received a saline solution (0.9%) i.p. and oral, and served as controls. The experimental design was a 2×2 factorial with two levels ACTH and two polyphenol levels (grape extract).

**Results:**

No significant differences were found in blood cortisol concentrations, by day and gender, or by treatment effects (0.75 µg/dL ± 0.11; *p *<0.001).

**Conclusion:**

Results suggest that chronic stress and consumption of resveratrol did not directly alter levels of plasmatic cortisol in either stressed or unstressed rats. It was concluded that the given dosage levels of ACTH possibly did not produce sufficient stimulation of the adrenal gland for these animals.

## Abstract (es)

**Objetivo:**

Determinar la actividad de cortisol en ratas tratadas con hormona adrecorticotropa (ACTH) exógena y un suplemento de resveratrol.

**Metodos:**

Se utilizaron 48 ratas hembras adultas y 16 machos de la cepa wistar (*Rattus norvegicus*) de tres meses de edad y con un peso corporal de 200-250 g y 300-350 g para hembras y machos, respectivamente, que permanecieron en condiciones ambientales controladas, temperatura 20±2° C de ciclos de luz-oscuridad de 14 y 10 horas, respectivamente. Se les proporcionó alimento balanceado con libre acceso a agua. Las ratas fueron divididas en cuatro grupos al azar así: grupo 1, fue tratado con 5 µg/kg de ACTH i.p. cada 12 horas; grupo 2, recibió el mismo tratamiento con ACTH además de una suplementación oral de 40 mg/kg de extracto deshidratado de uva (resveratrol); grupo 3, solo recibió extracto de uva y el grupo 4, recibió solución salina y sirvió como control y (0.9%) i.p. y oral. El diseño experimental fue en factorial 2×2, con dos niveles de ACTH y dos niveles de polifenol.

**Resultados:**

No se encontraron diferencias significativas del cortisol sanguíneo, con respecto al día y sexo, entre los tratamientos (0.75 µg/dL ± 0.11; *p* <0.001).

**Conclusión:**

Los resultados sugieren que el estrés crónico y el consumo de resveratrol no alteran directamente los niveles plasmáticos de cortisol, en ratas estresadas y no estresadas. De la misma manera que, posiblemente la dosis utilizada de ACTH no produjo estimulación de la glándula suprarrenal en las ratas.

## Introduction

The application of the physical term "stress" to the field of biological sciences is attributed to Hans Selye who discovered that stimuli could provoke this condition. This author defined stress as " the action of nervous and emotional stimuli caused by the environment on the nervous, endocrine, circulatory and digestive systems of an animal, producing measurable changes in functional levels of these systems"^1^. He also pointed out that stress has a positive relationship between the aggressiveness of the external environment and the magnitude of the organic response of the individual, i.e. defensive reactions to the stress-inducing agents which trigger functional responses capable of altering the regulatory mechanisms of homeostasis. It is well established that physiological and pathological consequences of exposure to stress depend on the characteristics of the stressful situation, as well as individual differences^2^. Stress triggers acute and chronic alterations in plasma concentrations of cortisol and thyroid hormones; it also may result in alterations in the physiological and behavioral reactions of animals^3^.

Cortisol is a corticosteroid produced by the adrenal gland; it is synthesized from cholesterol and its secretion is regulated by the adrenocorticotropic hormone (ACTH) produced in the pituitary gland, i.e. hypophysis^4^. The measurement of blood cortisol levels before and after exposure to the stressor indicates the individual´s response to biological stress^5^. Under conditions of metabolic stress associated with environmental and behavioral factors, the hypothalamic pituitary adrenal (HPA) axis stimulates the hypophysis to secrete more ACTH in comparison with normal conditions^6^.

Polyphenols (PFs) are a heterogenous group of chemical substances found in plants, which are characterized by the presence of an aromatic ring with hydroxyl groups, including esters and glycosides^7^. PFs are classified according to the number of carbons and phenol rings; also by the type and number of structural elements connected to the molecule (Table 1)^8^. Historically, some were considered anti-nutrients because they had the peculiarity of precipitating macromolecules such as proteins, carbohydrates and digestive enzymes, and reducing the digestibility of some foods. However, in the decade of the nineties there was increased interest in PFs for their possibly beneficial health effects. Beneficial effects were proposed for cardiovascular^9^ or neurodegenerative diseases^10^ and in the prevention and treatment of cancer^11^, and, in general, in those diseases in which oxidative stress might play an important role. These beneficial effects are mainly explained by the antioxidant, anti-inflammatory^12^ anti-cancerous^13^ properties of the PFs.

**Table 1:**
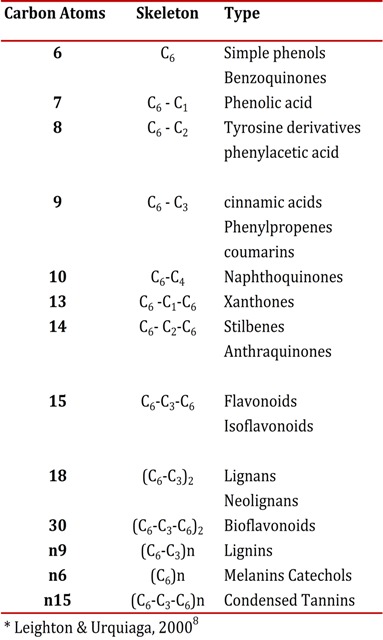
Plasma concentrations of cortisol in different treatment groups for rats subjected to chronic stress

The polyphenolic compounds of grapes (*Vitis vinifera*) are found in the skin, especially in the epidermal cells, and also in the seeds. The lowest concentrations found in the pulp. The quantity and quality of polyphenols in grapes depends on the variety of the vine, climate, terrain, and cultivation practices^14^. Of the components present in *Vitis vinifera*, the phenolic compounds, especially the proanthocyanidins, have attracted interest of the pharmaceutical industry. These natural antioxidants act as hijackers of free radicals; they are known to improve vasodilation and have anti-cancerous, anti-allergic, and anti-inflammatory properties while additionally stimulating the immune system. Finally, the estrogenic activity promotes the inhibition of enzymes including phospholipase A2, cyclooxygenase, and lipoxygenase, and stimulates others, such as superoxide dismutase^15^.

The object of this study was to determine the activity of cortisol in rats subjected to chronic stress supplemented with resveratrol.

## Materials and Methods

### Location: 

The experimental work was performed at the Laboratory for Nutrition and Poultry Health at the University of Caldas, Manizales (Caldas, Colombia). The laboratory relies on cages adapted to the species being investigated with appropriate food and water containers. Moreover, the laboratory offers a controlled environment.

### Animals: 

48 adult female rats were used and 16 males of the Wistar strain (*Rattus norvegicus*) that were three months of age with a body weight of 200-250 g for females and 300-350 g for males. They were kept in controlled environment with a temperature of 20 ± 2° C in light-dark cycles of 14 and 10 hours, respectively. The health of the animals was evaluated by clinical examination before the experimental period. The animals were randomly divided into four experimental groups of 12 females and four males each and were placed in four cages each composed of three females and one male. Each cage was taken as an experimental unit. All procedures of the experimental phase were carried out according to Colombian Legislation on Animal Care (Resolution 8030 of 1993) and approved by the Ethics Committee of the University of Caldas, Manizales, Colombia.

### Period of adaptation: 

The biomodels were from the vivarium (bioterio) of the Universidad del Valle, Cali, Colombia. The animals were transported for four hours in four 40-liter plastic boxes (57 cm long x 37 cm wide x 37 cm high). The animals had an adjustment period of ten days, at which time four random groups were formed each with 16 rats (12 females and four males) for treatment in four cages (three female and one male, each) and they were provided with commercial balanced feed (Rodentina, Agrinal Colombia SA) for rats (23.5% minimum protein; low fat 6.5%, maximum fiber 5% and maximum ash 8%), with free access to water.

### Treatments: 

Group 1 was treated with 5 µg/kg of ACTH intraperitoneal (ip) (Tetracosactrin acetate; Synacthen, Novartis(r), Barcelona, ​​Spain) every 12 hours (7:00 am to 7:00 pm) for 30-33 days. Group 2 received the same treatment with ACTH plus an oral supplement by gavage of 40 mg/kg of grape extract (Resverasor(r), Soria Natural SA Garray Soria, Spain). Group 3 received only grape extract 40 mg/kg. Group 4 served as a control and only received a saline solution (0.9%) i.p. and oral. Females were separated from the male by a glass that was removed 10 days after the adaptation period, and from that moment, every 12 hours, the presence or absence of a vaginal plug was visually evaluated for each to determine copulation, which was taken as day 0 of gestation.

### Sampling and analyses of cortisol: 

For measurement of plasma cortisol, 2 blood samples were taken in the morning (7:00 am) before treatment (day 0) and after treatment (days 30-33 and 23), at which time the animals were sacrificed, for females and males, respectively. Blood samples were taken by breaking the venous plexus of the ocular orbit (1 mL) with a heparinized capillary, with the animals briefly anesthetized with diethyl ether. At the end of the experimental period, the blood sample was obtained by cardiac puncture immediately after the sacrifice. Subsequently, the blood was put in vacutainer tubes, centrifuged at 1,300 × g for 5 minutes and the obtained plasma (0.2 mL) was stored in aliquots under freezing conditions at -20° C until processing.

The blood concentration of cortisol (µg/dL) was determined with the electrochemical luminescence technique (Advia Centaur Siemens). It consists of chemical reactions in which a chemiluminescent precursor is treated with oxidizing agents and catalysts to produce an intermediate product which when electronically excited produces electromagnetic radiation in a visible photon spectrum. The analytical sensitivity was 0.018 μg /dL and had a functional sensitivity of 0.07 μg/dL. The measurements were performed in the Comfamiliares Clinical Laboratory in Manizales, Caldas, Colombia. The samples were taken from one female and one male per cage (experimental unit) for each of the treatments. 

### Experimental design and statistical analysis: 

The design was experimental, randomly balanced and complete, with an allocation of treatments in a 2 × 2 factorial arrangement with two levels of ACTH (absence and presence) and two levels of polyphenol (absence and presence), and with four replications. The experimental unity consisted of a cage with three females and one male. By means of an analysis of variance and the effect of ACTH and polyphenol and their linear interaction on cortisol was evaluated. Also, the day effect on the concentration of cortisol was evaluated.

## Results and Discussion

From the results obtained, no significant differences in the concentrations of the cortisol, regarding the day and treatment (*p*> 0.05) (Table 2) were found.

**Table 2:**
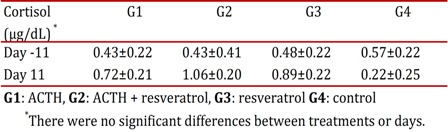
plasma concentrations of cortisol in the different treatments in rats subjected to chronic stress. G1: ACTHG2: ACTH + G3 resveratrol: resveratrol G4: control. there were no significant differences between treatments and days

Ulrich-Lai *et al*., have described the positive regulation of ACTH on the production of glucocorticoids by the adrenal glands in rats^16^. ACTH stimulates the secretion of glucocorticoids and elevates intracellular levels of AMPc, which quickly induces an increase in the activity of the Steroidogenic Acute Regulatory (StAR) enzyme which increases the influx of cholesterol to the mitochondria. On the other hand, ACTH increases the activity of other enzymes, such as cytochrome P450, involved in the synthesis of glucocorticoids and contributes to a net increase in production. Plasma levels of glucocorticoids act directly on the pituitary or on hypothalamic neurons and exert a negative feedback on their secretion, protecting the organism from the effects of hypercortisolism^17^.

Circulating glucocorticoids have circadian rhythms that are characterized by peak activity in the early morning hours of the day for diurnal animals, including humans, and during evenings for nocturnal animals^18^. The rhythm may be endogenous or exogenous as generated by the organism itself, although it has been reported that it is only considered a rhythm when it is endogenous^19^. Endogenous rhythms are produced by a biological clock that in mammals is located in the suprachiasmatic nucleus of the hypothalamus. Because of its endogenous origin, these rhythms are expressed even in the absence of external environmental cycles. According to their frequency, circadian rhythms are classified as those having a frequency close to one day, i.e. between 22 and 28 hours. However, it should be mentioned that superimposed on circadian rhythms are rhythms of greater frequency, referred to as ultradian, that corresponds to the pulsatile secretion of certain hormones (every 2 hours), as observed with cortisol, that in addition to the ultradian rhythm, follows a circadian rhythm of approximately 24 hours^19^.

In hamsters, lesions on the suprachiasmatic nucleus suppress the circadian rhythm of cortisol and corticosterone (both steroids are synthesized by the adrenal cortex in this species), which generates a high dispersion of plasma levels for these hormones^20^. This set of results suggests that the circadian rhythm of secretion of glucocorticoids by the adrenal cortex is regulated not only for the control of the suprachiasmatic nucleus of the plasma levels of ACTH, but also by way of direct innervations as demonstrated by Buijs *et al* ^20^. However, these results do not exclude an intrinsic ability of the adrenal cortex to produce rhythmic functions.

Both the circadian rhythm of glucocorticoids and the sensitivity of the response of the adrenal cortex to ACTH depend on the suprachiasmatic nucleus. In rats with previous electocoagulation lesions in the central nervous system, treatment with dexamethasone, which inhibits endogenous ACTH, indicated changes in the sensitivity of the adrenal gland. However, in rats treated with dexamethasone, differences in the magnitude of the response to ACTH were observed when the animals were grouped according to the basal corticosterone levels (38-373 ng/mL), which could mean that the adrenal gland maintains its intrinsic ability to respond differentially to ACTH^21^. According to some authors^22^, and contrary to what was reported by Ulrich-Lai *et al*.^16^, in rats with an intact suprachiasmatic nucleus the morning/afternoon difference in sensitivity to ACTH disappears when the adrenal gland is enervated.

Taking the above into account, the time of day at which blood samples were taken for cortisol measurement can be a critical factor due to the circadian rhythm that the hormone presents. Unlike findings from other studies that did not specify the time of day when the sample was taken, in the present study this was not a factor of variation from the results obtained by Radahmadi *et al*. ^23^, who reported that cortisol levels were determined 14 days after stress was generated in the rats (diabetics with and without stress, and non-diabetics with and without stress). No alterations were presented as the adrenal gland can adapt to the stimulus that stress produces. Therefore, when there is a repeated stressor, the adrenal gland may not be able to respond possibly due to the adaptation to the physical stress.

Glucocorticoids are believed to be crucial for the responses at the beginning and end of stress. It appears that the role of glucocorticoid receptors is exerted in the last phase of the response, and results in recovery from the initial stress to a state of normality for the organism. In this last phase, storage occurs for the memory of what happened, allowing the body to prepare the organism for similar new emergency situations. In addition, energy resources are mobilized to prepare for future events, also restoring the previously altered homeostasis in the initial phase for the output of glucocorticoids^24^. Therefore, chronic stress can cause an increase in the secretion of ACTH that compensates for negative cortical feedback and the stored memory possibly generated by previous experience.

Chronic stress increases oxidative stress through increased production of reactive oxygen species^25^. In animals subjected to oxidative stress conditions, the different biological systems attacked give off a warning signal from sympathetic nerves that transforms through a series of metabolic and endocrine stages in glucocorticoids^26^. Antioxidants are intended to reduce the toxic effect and control the origin of pathologies caused by oxidative stress. Therefore, when oxidative stress is met with an antioxidant product such as resveratrol, the levels of glucocorticoids should possibly decrease.

Because the rats reached a stage of resistance to stress, i.e. that the animals faced the presence of the threatening factor with ACTH, possibly to the antioxidant action and it is at this stage where the hypothalamic-hypophysis axis participates and to the adrenal cortex normalization occurs for levels of glucocorticoids.

Applying ACTH every 12 hours did not produce change in plasma concentrations of cortisol among the four treatment groups. However, the manipulation of the animals could create an adrenal adaptation, probably why no changes were observed in the concentration of plasma cortisol.

## Conclusion

The results suggest that the chronic stress induced by ACTH and the consumption of resveratrol does not directly alter plasma cortisol levels in treated and untreated rats. Similarly, the ACTH dose used did not produce stimulation of the adrenal gland in rats.
